# Supplementary material for: Healing Properties of Epidermal Growth Factor and Tocotrienol-Rich Fraction in Deep Partial-Thickness Experimental Burn Wounds
Source: Antioxidants (Basel). 2020 Feb 3;9(2):130. doi: 10.3390/antiox9020130 (PMC7070471; doi:10.3390/antiox9020130)
Supplement: Supplementary file 1 [file antioxidants-09-00130-s001.pdf]

Supplementary files

Table S1. Description of histological score 0 for the epidermis.

<table>
<tr><td>Score</td><td colspan="3">0</td></tr>
<tr><td rowspan="2">Description</td><td>Crust</td><td>Epithelialization</td><td>Rete ridge</td></tr>
<tr><td>Loosely attached</td><td>No</td><td>No</td></tr>
<tr><td>Image
(40×)</td><td colspan="3">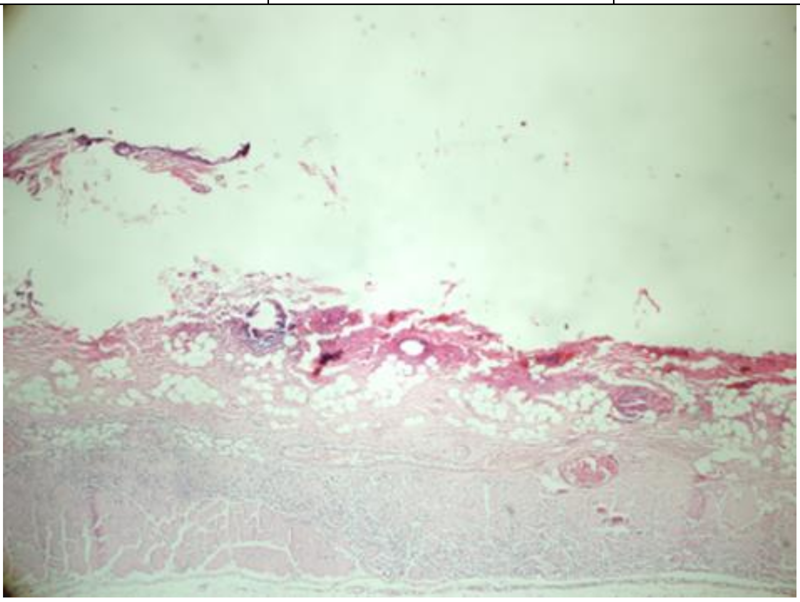</td></tr>
<tr><td>Image
(100×)</td><td colspan="3">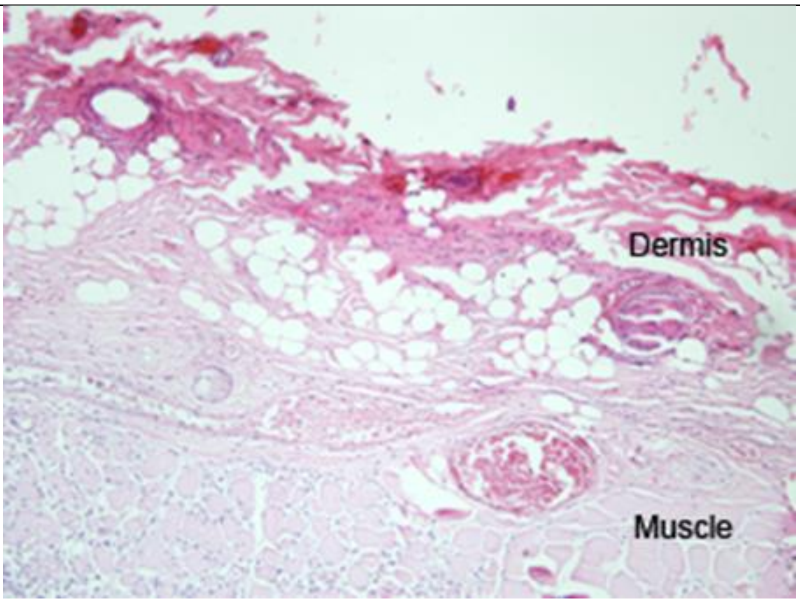
</td></tr>
</table>

Table S2. Description of histological score 1 for the epidermis

Score	1		
Description	Crust	Epithelialization	Rete ridge
	Tightly attached	Minimal	No
Image (40×)	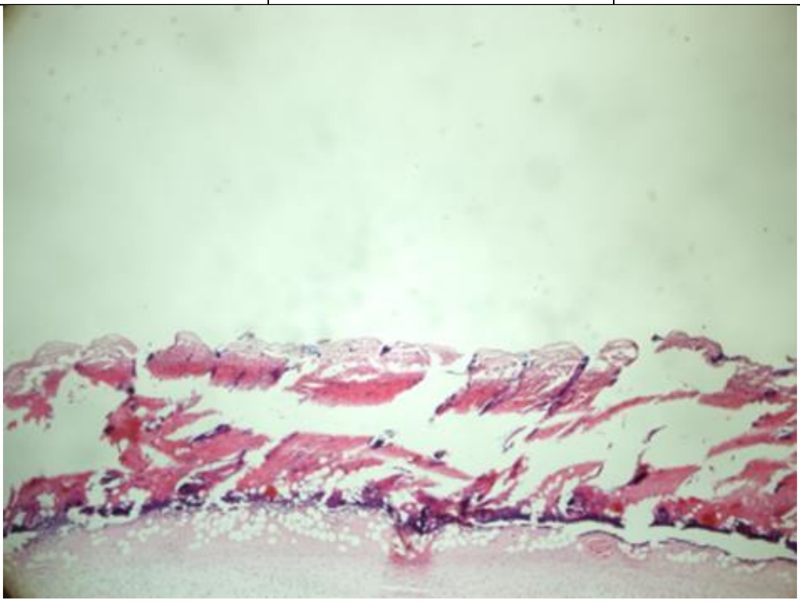		
Image (100×)	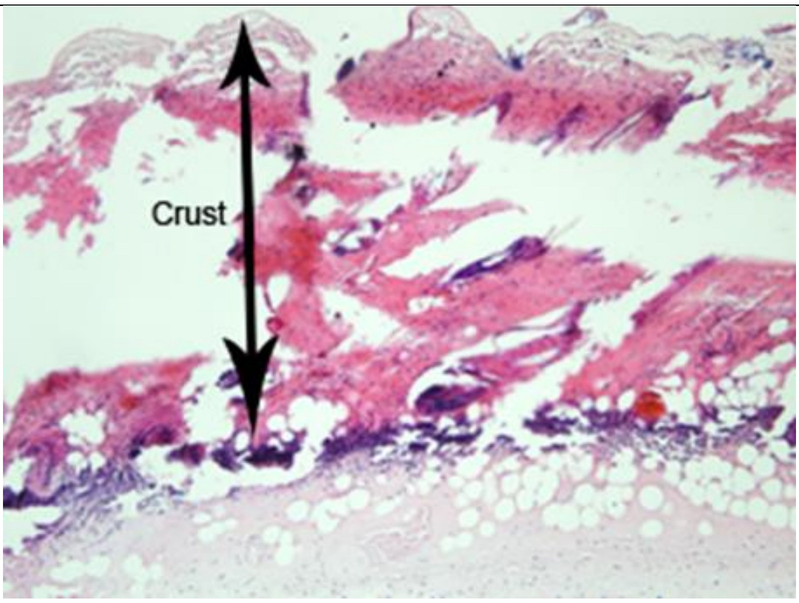		

Table S3. Description of histological score 2 for the epidermis

Score	2		
Description	Crust	Epithelialization	Rete ridge
	Tightly attached	Mild	No
Image (40×)	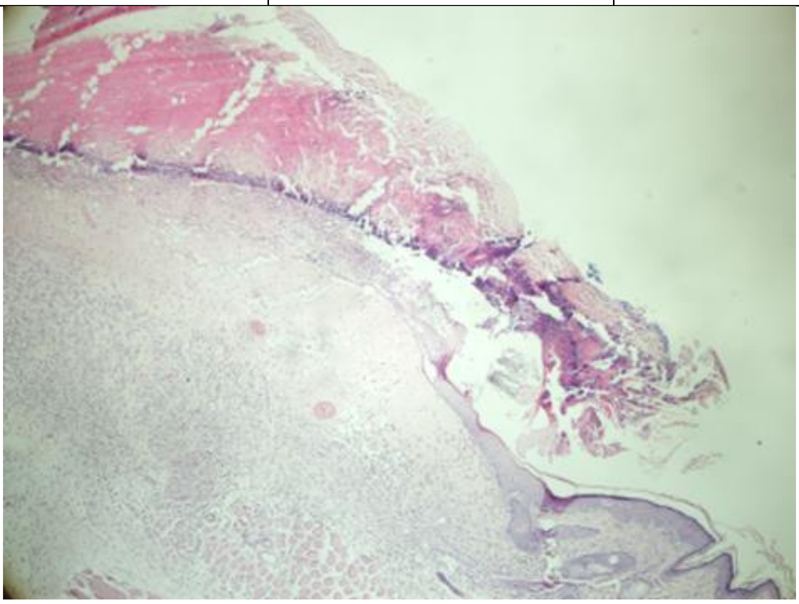		
Image (100×)	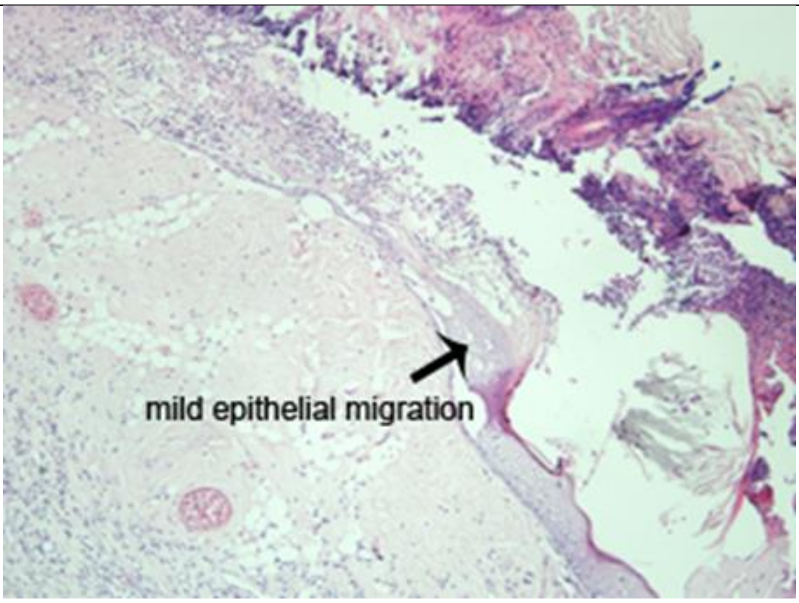		

Table S4. Description of histological score 3 for the epidermis

Score	3		
Description	Crust	Epithelialization	Rete ridge
	Tightly attached	Moderate	No
Image (40×)	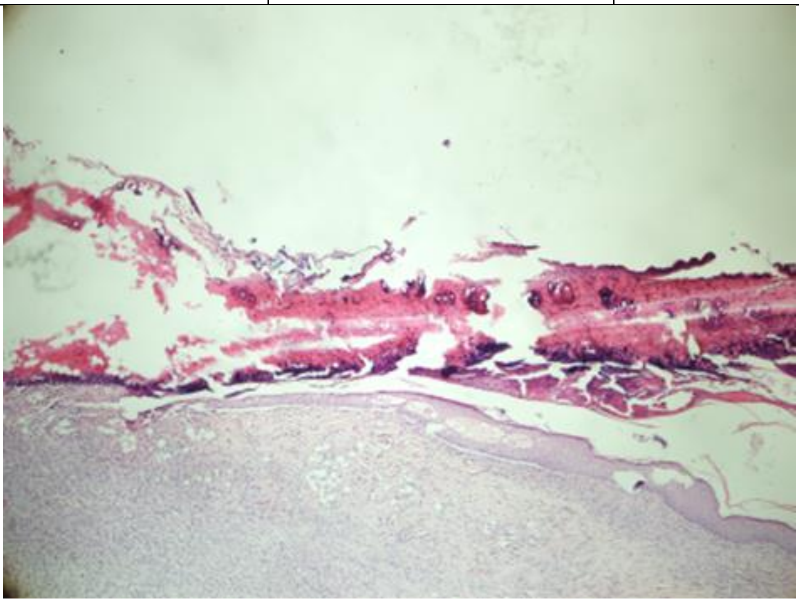		
Image (100×)	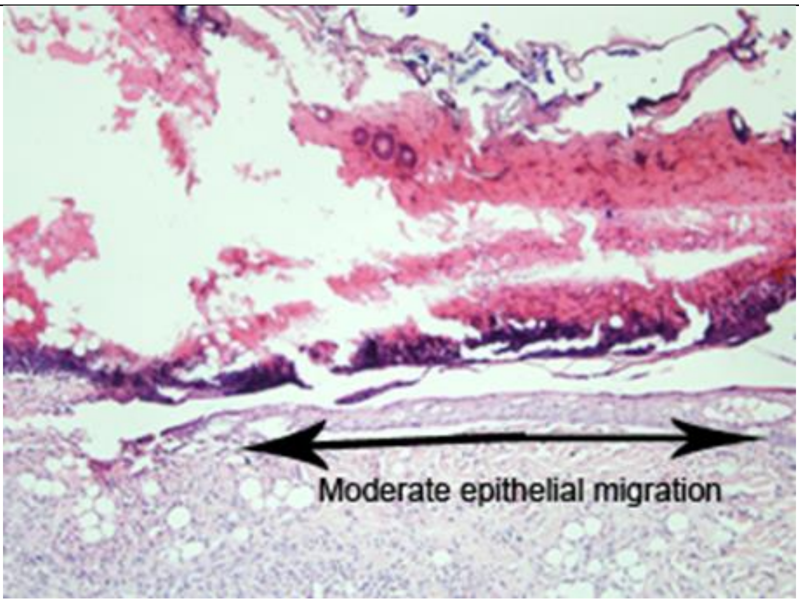		

Table S5. Description of histological score 4 for the epidermis

Score	4		
Description	Crust	Epithelialization	Rete ridge
	No	Moderate	No
Image (40×)	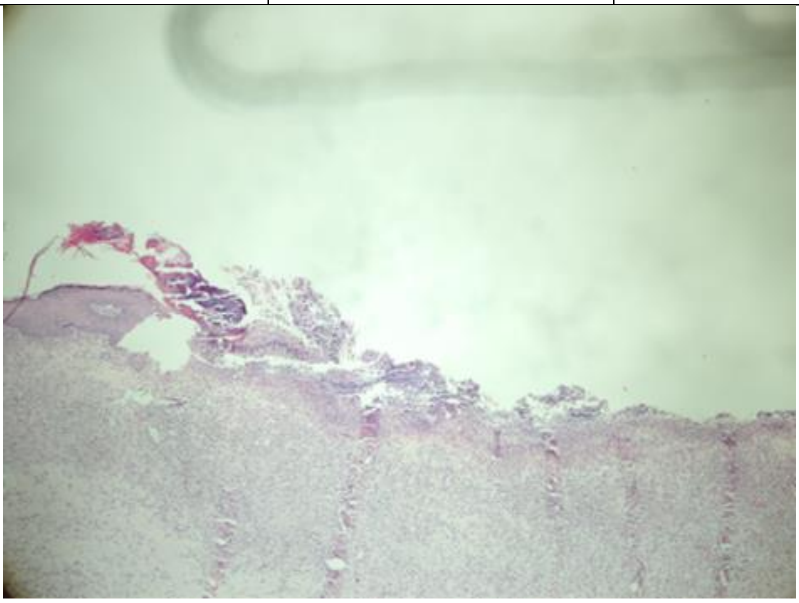		
Image (100×)	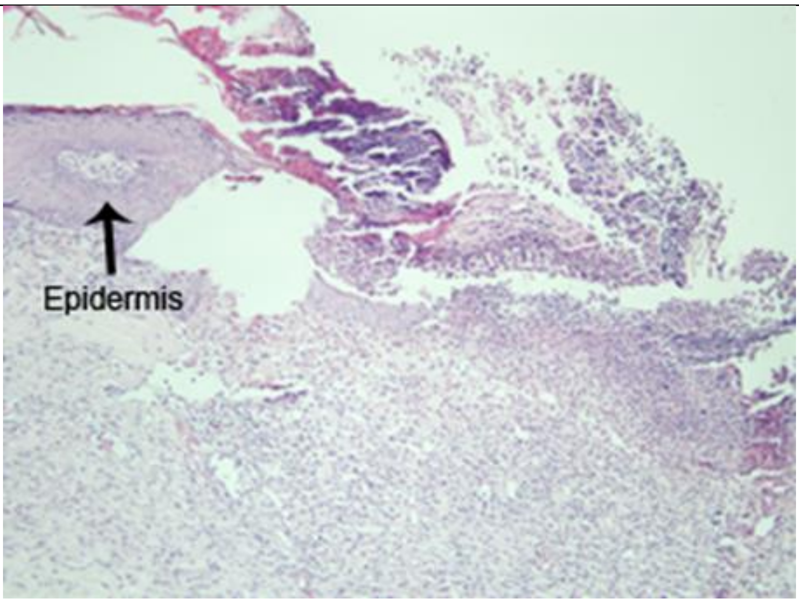		

Table S6. Description of histological score 5 for the epidermis

Score	5		
Description	Crust	Epithelialization	Rete ridge
	No	Severe	No
Image<br>(40×)	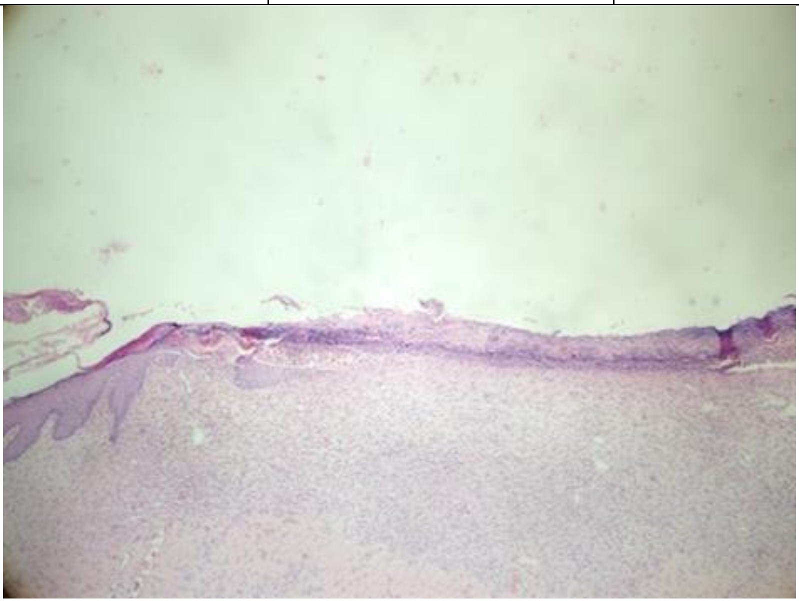		
Image<br>(100×)	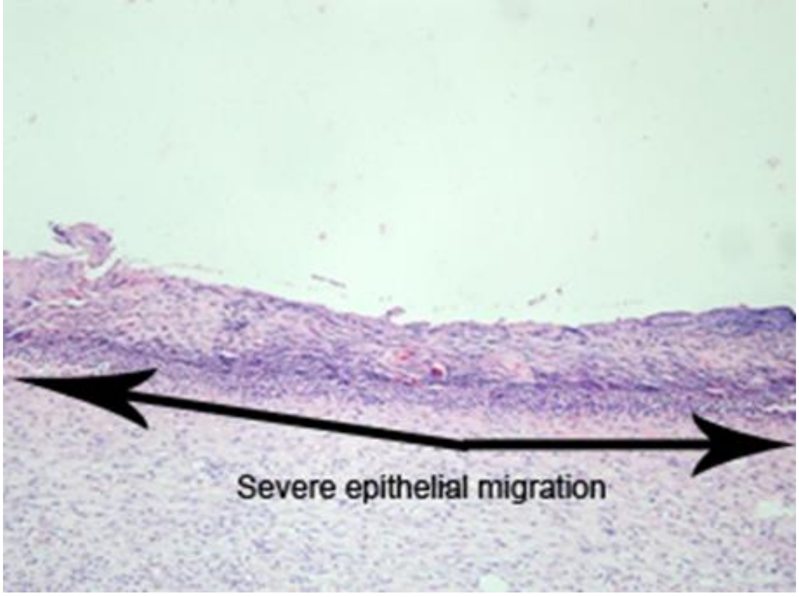		

Table S7. Description of histological score 6 for the epidermis

Score	6		
Description	Crust	Epithelialization	Rete ridge
	No	Complete	No
Image (40×)	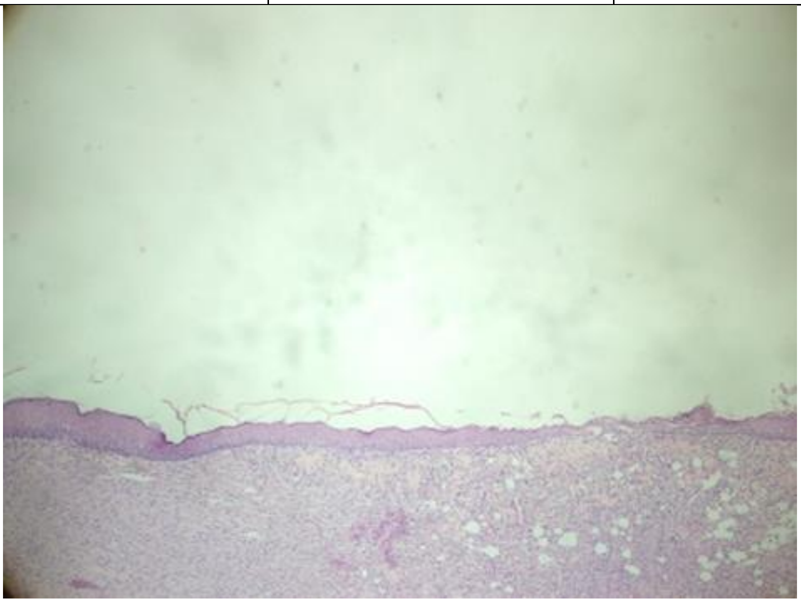		
Image (100×)	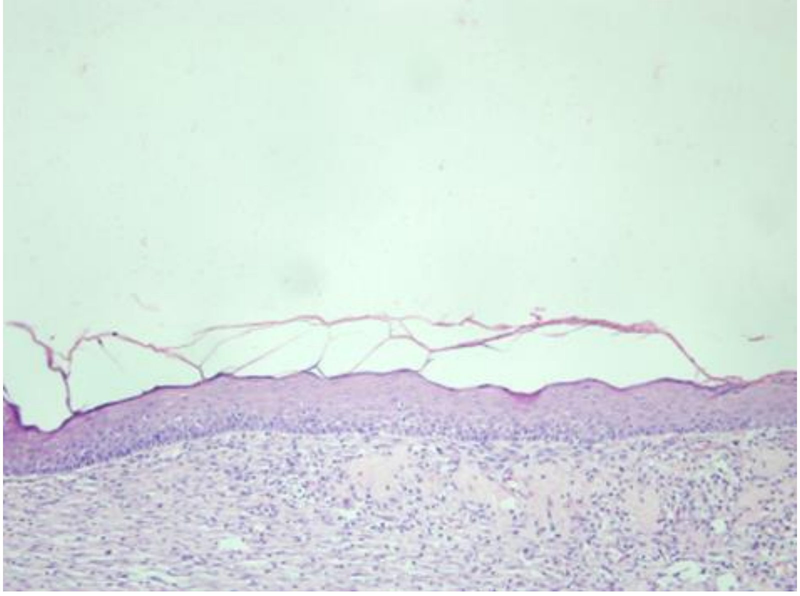		

Table S8. Description of histological score 7 for the epidermis

<table>
<tr><td>Score</td><td colspan="3">7</td></tr>
<tr><td rowspan="2">Description</td><td>Crust</td><td>Epithelialization</td><td>Rete ridge</td></tr>
<tr><td>No</td><td>Complete</td><td>Yes</td></tr>
<tr><td>Image
(40×)</td><td colspan="3">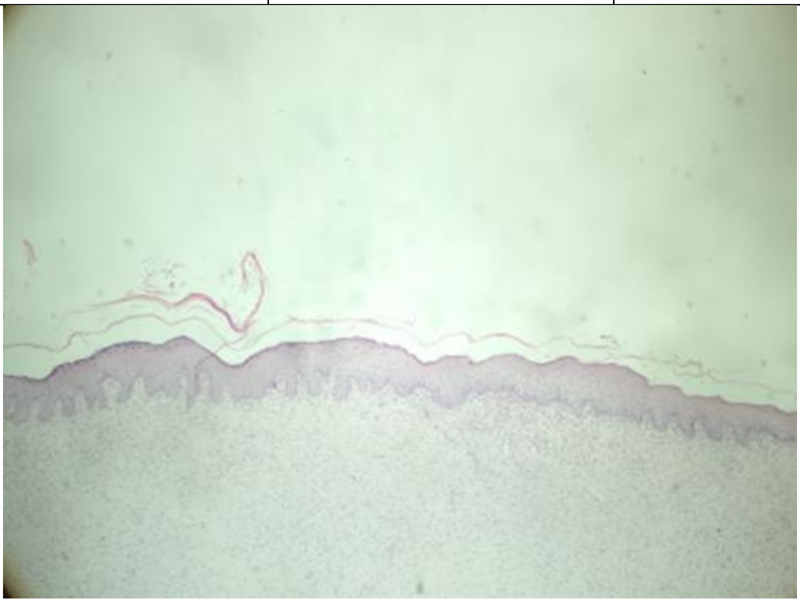</td></tr>
<tr><td>Image
(100×)</td><td colspan="3">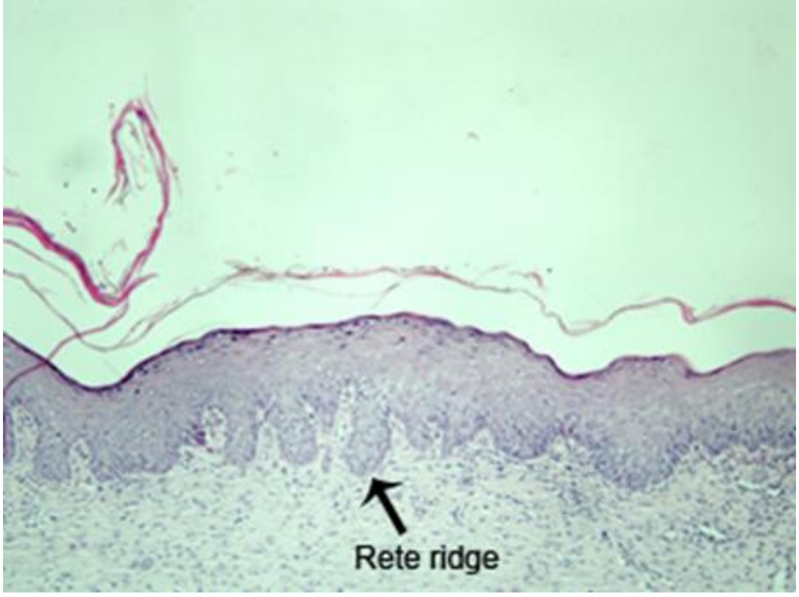
</td></tr>
</table>

3.3.2.3.2 Histological score grade for dermis

According to the proportion of adipose cells, inflammatory cells and fibroblasts, the state of collagen deposition, and the formation of hair follicles, the healing of the dermis was also evaluated with scores ranging from 0 - 7 (Table 3.12 and Table 3.13). The following is a detailed explanation of each score:

0: Many adipose cells; A few inflammatory cells; No fibroblasts; No collagen deposition; No formation of hair follicles.

1: Medium adipose cells; Medium inflammatory cells; A few fibroblasts; No collagen deposition; No formation of hair follicles.

2: A few adipose cells; Medium inflammatory cells; A few fibroblasts; No collagen deposition; No formation of hair follicles.

3: A few adipose cells; Many inflammatory cells; A few fibroblasts; No collagen deposition. No formation of hair follicles.

4: A few adipose cells; Many inflammatory cells; Medium fibroblasts; mild collagen deposition. No formation of hair follicles.

5: No adipose cells; Medium inflammatory cells; Many fibroblasts; Moderate collagen deposition; No formation of hair follicles.

6: No adipose cells; A few inflammatory cells; Many fibroblasts; Moderate collagen deposition. No formation of hair follicles.

7: No adipose cells; A few inflammatory cells; Medium fibroblasts; Severe collagen deposition. The immature hair follicles were observed.

Table S9 Histological score range for the dermis.

score	Adipose cell	Inflammatory cell	Fibroblast	Collagen deposition	Hair follicle
0	+++	+	-	-	-
1	++	++	+	-	-
2	+	++	+	-	-
3	+	+++	+	-	-
4	+	+++	++	+	-
5	-	++	+++	++	-
6	-	+	+++	++	-
7	-	+	++	+++	+

Note: -: absent +: mild ++: moderate +++: severe

Table S10. Images of histological score for the dermis.

Score	Description	Image (100x)
0	Many adipose cells; A few inflammatory cells;	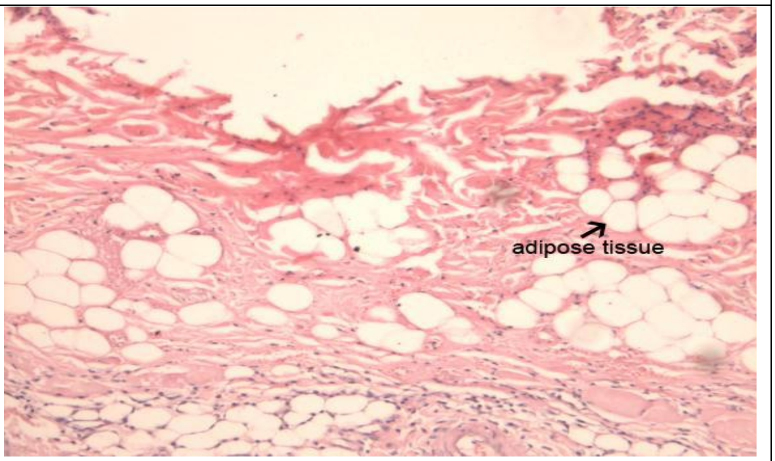
1	Medium adipose cells; Medium inflammatory cells; A few fibroblasts;	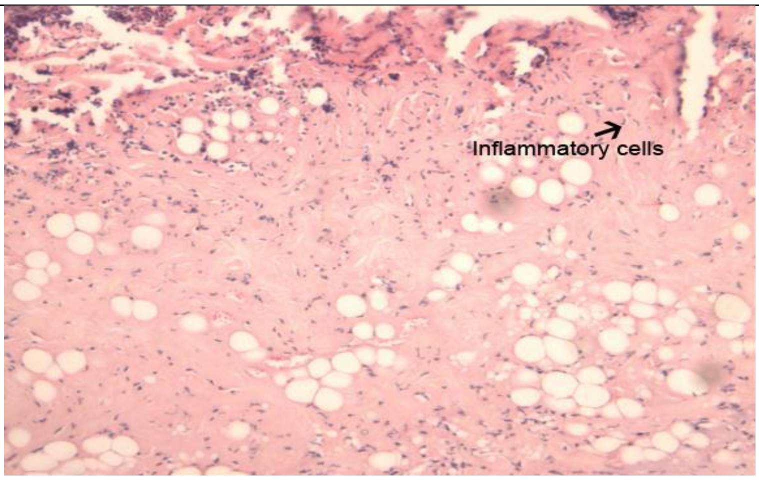
2	A few adipose cells; Medium inflammatory cells; A few fibroblasts;	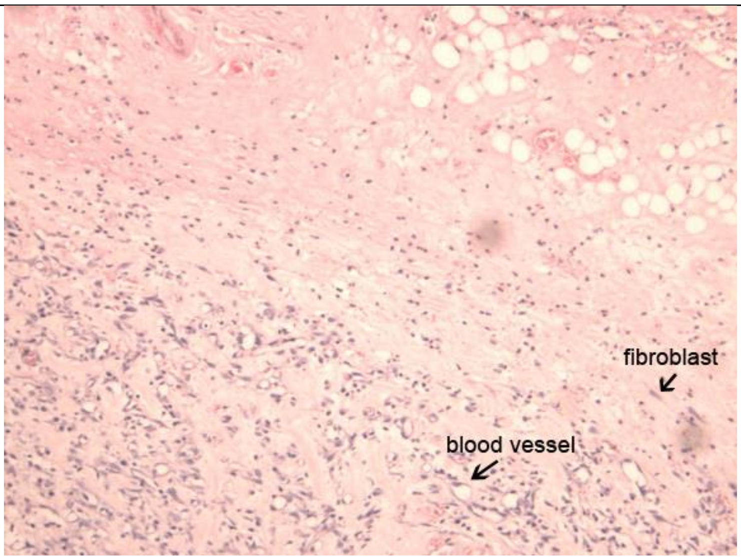

3	A few adipose cells; Many inflammatory cells; A few fibroblasts;	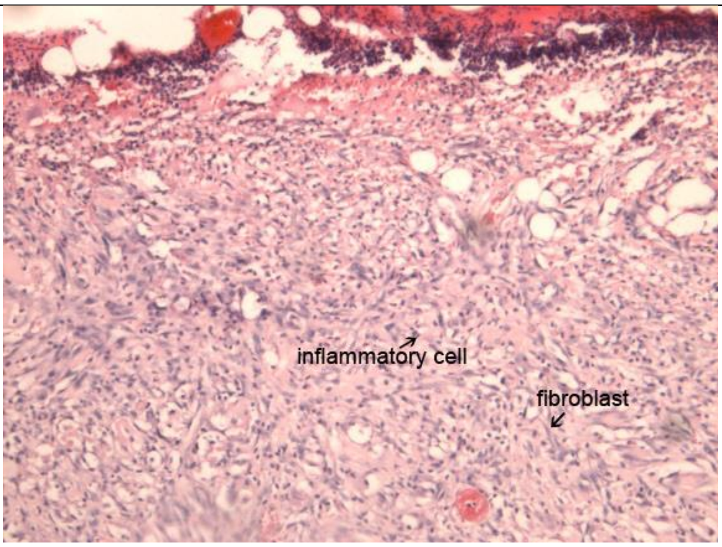
4	A few adipose cells; Many inflammatory cells; Medium fibroblasts; mild collagen deposition	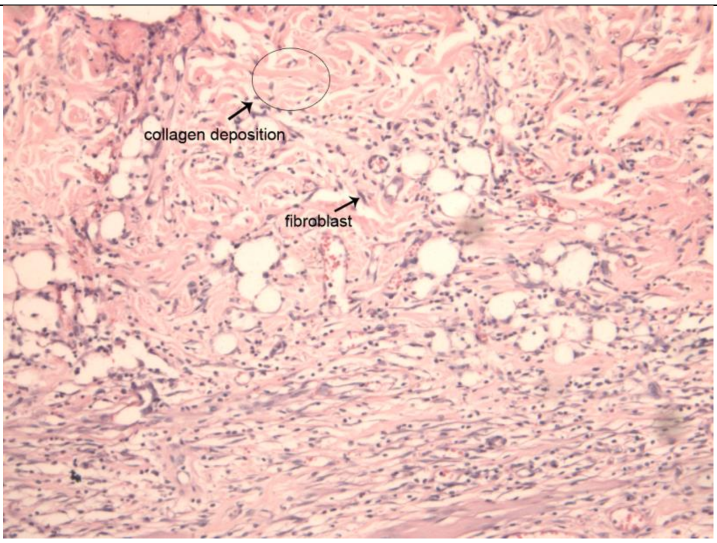
5	Medium inflammatory cells; Many fibroblasts; Moderate collagen deposition;	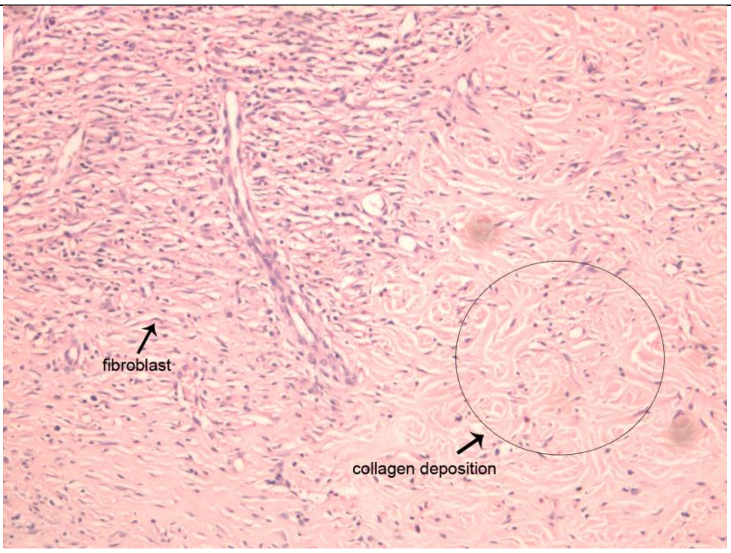

6	A few inflammatory cells; Many fibroblasts; Moderate collagen deposition.	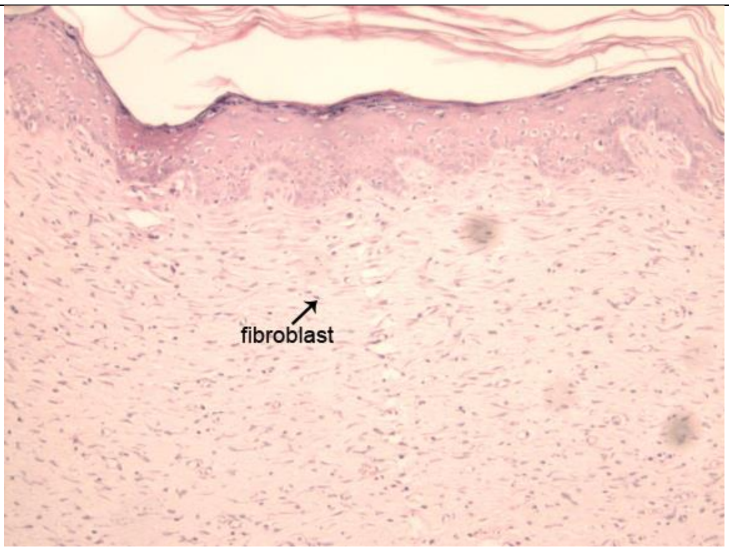
7	A few inflammatory cells; Medium fibroblasts; Severe collagen deposition. The immature hair follicles were observed.	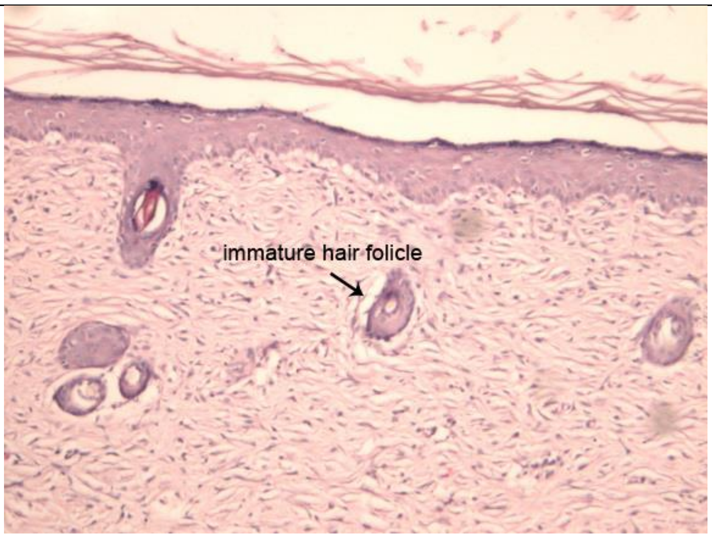
